# Biserial Miyaguchi–Preneel Blockchain-Based Ruzicka-Indexed Deep Perceptive Learning for Malware Detection in IoMT

**DOI:** 10.3390/s21217119

**Published:** 2021-10-27

**Authors:** Abdullah Shawan Alotaibi

**Affiliations:** Department of Computer Science, College of Science and Humanities Al Dawadmi, Shaqra University, Shaqra 11961, Saudi Arabia; a.shawan@su.edu.sa; Tel.: +966-533-454-270

**Keywords:** malware detection, internet of medical things (IoMT), deep multilayer perceptive learning, point biserial correlation, Miyaguchi-Preneel cryptographic hash-based blockchain, Ruzika index

## Abstract

Detection of unknown malware and its variants remains both an operational and a research challenge in the Internet of Things (IoT). The Internet of Medical Things (IoMT) is a particular type of IoT network which deals with communication through smart healthcare (medical) devices. One of the prevailing problems currently facing IoMT solutions is security and privacy vulnerability. Previous malware detection methods have failed to provide security and privacy. In order to overcome this issue, the current study introduces a novel technique called biserial correlative Miyaguchi–Preneel blockchain-based Ruzicka-index deep multilayer perceptive learning (BCMPB-RIDMPL). The present research aims to improve the accuracy of malware detection and minimizes time consumption. The current study combines the advantages of machine-learning techniques and blockchain technology. The BCMPB-RIDMPL technique consists of one input layer, three hidden layers, and one output layer to detect the malware. The input layer receives the number of applications and malware features as input. After that, the malware features are sent to the hidden layer 1, in which feature selection is carried out using point biserial correlation, which reduces the time required to detect the malware. Then, the selected features and applications are sent to the hidden layer 2. In that layer, Miyaguchi–Preneel cryptographic hash-based blockchain is applied to generate the hash value for each selected feature. The generated hash values are stored in the blockchain, after which the classification is performed in the third hidden layer. The BCMPB-RIDMPL technique uses the Ruzicka index to verify the hash values of the training and testing malware features. If the hash is valid, then the application is classified as malware, otherwise it is classified as benign. This method improves the accuracy of malware detection. Experiments have been carried out on factors such as malware detection accuracy, Matthews’s correlation coefficient, and malware detection time with respect to a number of applications. The observed quantitative results show that our proposed BCMPB-RIDMPL method provides superior performance compared with state-of-the-art methods.

## 1. Introduction

The Internet of Things (IoT) is an extensive network of smart, internet-connected devices that automatically sense and collect data from various environments. With the rapid growth of IoT technologies, the detection and analysis of malware is a significant concern in medical application systems. Malicious applications may damage android IoT devices. Therefore, increased attention must be paid to the detection of these applications to stem privacy leakage and property loss. Several methods have been developed to detect malware, which are discussed here.

A highly scalable malware detection framework, enabled for software-defined networking (SDN), which was a hybrid convolutional neural network with long short-term memory (CNN-LSTM), was developed for the Internet of Medical Things (IoMT) [1]. LSTM was employed to handle the long-term dependency. Binary classification was used to predict malware attack by reducing the low computational complexity. The designed framework increased the accuracy of malware detection but did not reduce the time consumption from that required in previous methods. A multi-kernel support vector machine (SVM) was also developed for IoT malware hunting [2]. The design approach used the grey wolf optimization (GWO) technique for feature selection. The feature selection module was introduced to minimize the number of features and the computational cost. The multi-kernel SVM classifier was applied for precisely determining the IoT malware. However, it did not incorporate efficient machine-learning algorithms that could improve the accuracy of the search for malware threats.

Kumar et al. (2019) devised a novel method that integrated machine-learning techniques and blockchain to detect malware in Android IoT devices [3]. The malware information was extracted with aid of the Machine learning method. This information was stored in the blockchain. The classification algorithm was used to attain maximum accuracy with a naive Bayes classifier for addressing the multi-feature issue. However, the method failed to use the deep neural network for malware detection using blockchain to attain more security and privacy. A modified two-hidden-layered extreme learning machine (TELM) was also developed to detect malware with higher accuracy [4]. The bagging-based ensemble method was employed to stable the model. The designed TELM was enhanced for classification performance, but again the time consumption required due to the complexity of malware detection was not minimized.

Jahromi et al. (2020) introduced a stacked LSTM method that improved malware detection performance [5]. The designed LSTM method was used to enhance the accuracy, but the efficient feature selection method was not coupled with measures to reduce time consumption. Ren et al. (2020) developed an end-to-end Android malware detection approach that was based on deep learning with minimum resource utilization [6]. The resampling method was applied to pre-process the classes.dex file in Android APK. The two end-to-end Android malware detection methods were applied to obtain the detection accuracy. However, this method did not include a larger, production-scale dataset for malware detection. 

Two different methods were developed for malware detection. The Robust-NN was designed with the integration of a convolutional neural network and 1- nearest neighbours (C4N) for enhancing the accuracy [7]. However, these deep-learning methods did not offer dynamic features that could improve their performance against adversarial attacks. A novel method based on hash results to detect malware increased the true detection rate but did not reduce the time required for the job [8].

Takase et al. (2020) used values that were extracted from the processor to detect malware. The designed mechanism was used to categorize malware or benign programs with the aid of processor information to improve security, but no hash-based malware feature analysis was performed to minimize the time consumption [9]. Finally, dynamic analysis for the IoT malware detection (DAIMD) system was developed for malware detection [10]. The designed system includes debugging, feature extraction, feature pre-processing, feature selection, and classification for identifying malware or benign programs. However, the system did not use the dimensionality reduction technique effectively for accurate malware detection.

### 1.1. Research Gap

Malware detection is one of the most important threats in IoT. Machine learning is one of the most effective methods employed to detect malware in an IoT environment. Previous work has shown that the malware detection method has not yet solved the two-fold concern that malware must be detected accurately but as quickly as possible. Previous studies have shown that this method takes more time to classify malware detection and does not possess the expertise to effectively and timely handle the massive numbers of newly discovered malware. To overcome this issue, the current BCMPB-RIDMPL technique has been introduced.

### 1.2. Objective

The objective of the proposed technique is as follows:

- To enhance malware application detection with lesser time consumption, the BCMPB-RIDMPL technique is introduced;
- To minimize the malware detection time, the BCMPB-RIDMPL technique uses Point biserial correlative feature matching to select the relevant feature and remove the unnecessary malware features;
- To generate the hash value with maximum security, the Miyaguchi–Preneel cryptographic hash-based Blockchain is applied in the BCMPB-RIDMPL technique;
- To match the hash value, the Ruzicka similarity index is applied for categorizing the malware and benign applications in BCMPB-RIDMPL.

### 1.3. Contribution of This Study

The present work explains the development of a novel biserial correlative Miyaguchi–Preneel blockchain-based Ruzicka-index deep multilayer perceptive learning (BCMPB-RIDMPL) technique. The research contributions described in this paper are summarized here:

- The BCMPB-RIDMPL technique is shown to improve the detection of malware applications through the choice of the processes of feature selection, blockchain technology, and classification;
- The method is shown to match the point biserial correlative feature to select the relevant feature and remove the malware;
- The novelty of the point biserial correlation coefficient is employed to estimate the correlation among the features. The maximum correlations among the features are chosen and other features are eliminated. It is used for minimizing the dimensionality of data and malware detection time;
- The Miyaguchi–Preneel cryptographic hash-based blockchain is applied to create the hash value for each selected feature;
- The novelty of the blockchain technology Miyaguchi–Preneel compression function is used for creating the hash value. This helps to ensure security by avoiding unauthorized access;
- The novelty of the Ruzicka similarity index is used to verify the hash value of malware and other test features and classifies malware and benign applications;
- Lastly, the paper explains the extensive experimental evaluations that have been performed in various performance metrics to highlight the benefit of the proposed BCMPB-RIDMPL method over conventional classification techniques.

### 1.4. Organization of Paper

The article is organised into five different sections as follows. Section 2 reviews the related works in malware detection; the proposed BCMPB-RIDMPL technique is described in Section 3 with a diagram; Section 4 presents the experimental settings with the dataset description; Section 5 discusses the analysis of the experimental results with different performance metrics; and finally, Section 6 provides the conclusion of the proposed method. 

## 2. Related Work

Das et al. (2019) analyzed the different types of malware detection approaches that have been applied to improve the security of the IoT/IoMT environment [11]. Different architectures of the IoT environment and their applications are examined. A taxonomy of security schemes in an IoT/IoMT environment was offered but failed to secure communication by using a blockchain-based malware detection scheme. Imtiaz et al. (2021) developed a deep artificial neural network (ANN) that improved the efficiency of real-world Android malware detection [12]. The designed approach was used to categorize the malware attacks instatic and dynamic layers. However, it did not involve an online service through which a user was able to find a benign or malicious application before downloading it.

An advanced ensemble learning (MTHAEL) method was developed for robust IoT malware threat detection [13]. The designed MTHAEL method improves the accuracy and reduces the computation overhead. However, the method does not apply large-scale IoT applications or multi-class predictions to improve the classification techniques. A deep recurrent neural network (RNN) learning model was developed to detect threats to cryptocurrency. The text-mining method was employed to obtain the optimum features set from the extracted sequence of the opcode. However, it failed to meet the expected detection accuracy [14].

Lei et al. (2019) developed a scalable and event-aware android malware detection method. The neural network was used for extracting high-level semantics. The softmax classifier was employed to create the prediction, but the deep-learning model was not implemented for accurate malware detection [15]. A sub-curve hidden Markov model was developed for feature extraction to identify malware [16]. The designed model was used to discover discontinuities in the series of probability scores, called Sub-Curve. The classifiers are used to detect fragments of malware’s suspicious behaviors. However, it did not perform the data pre-processing that was required for accurate extraction of malicious application programming interface (API) sequences to increase the efficiency of the detection of malware fractions.

Guizani and Ghafoor (2020) identified a software-based architecture for malware detection to improve the security performance of IoT devices [17]. The designed architecture was used to predict malware attacks for enhancing the feasibility. However, the authors did not apply the machine-learning technique to improve the accuracy and minimize the false-positive rate. Lu et al. (2021) followed this work with a new malware detection and analysis scheme based on the CNN deep-learning classifier [18]. The dissimilar loss function was employed for attaining the multiclass classification. The learning performance was improved. However, the amount of time that was needed for malware detection remained unaddressed.

A machine-learning-based visualization scheme that was developed by Liu et al. (2020) for malware identification could not be applied to a wide range of applications in machine learning and computer security [19], but the false positive rate was not considered. A recurrent neural network model was introduced to identify IoT and networking malware threads. Long-Short-Term-Memory was used for enhancing network security, but it could not be applied to select a significant number of features [20]. Bolton et al. (2021) investigated the security and privacy issue in virtual assistants [21]. The voice assistants were an important part of detecting malicious attacks. The study was performed to enhance user authentication performance. 

A deep learning-based ensemble learning detection framework was introduced by Ma, et al. (2019) for API fragments [22], but the malware detection accuracy was not improved. Xiao, et al. (2019) developed a novel behavior-based deep learning framework (BDLF) to classify the malware attack and increase the precision [23]. However, the malware detection and classification were not focused. A machine-learning model with SDN-enabled security was introduced by Haseebet al. (2021) to enhance network consumption [24]. Anand et al. (2021) introduced a deep learning model (CNN-DMA) for discovering malware attacks [25]. Decentralised blockchain-enabled privacy-preserving trajectory data mining framework was presented by Talat, R al. (2020) to find the mobility patterns in privacy preserving [26]. The designed framework was employed to improve the scalability and reduce the execution time. 

## 3. Methodology of Current Work

The IoMT is a network of connected wearable medical devices and medical systems. It facilitates a variety of healthcare services to minimize healthcare expenditure, offers timely medical responses, and increases the quality of medical treatment. In recent years, numerous works have been designed for malware detection. Accurate malware detection is a challenging task, since the machine learning techniques use the entire feature for classification, therefore making it infeasible and inaccurate. Hence, the dimensionality of the dataset needs to be minimized for accurate malware detection with minimum time consumption. In order to overcome the issue, a BCMPB-RIDMPL technique is introduced. The proposed technique aims to enhance the accuracy of malware detection with minimum time consumption and false-positive rate. The BCMPB-RIDMPL technique performs feature selection and classification, using Point biserial correlative feature matching to identify the relevant features for accurate detection. The selected relevant features are used to classify and detect the malware.

**Figure 1:**
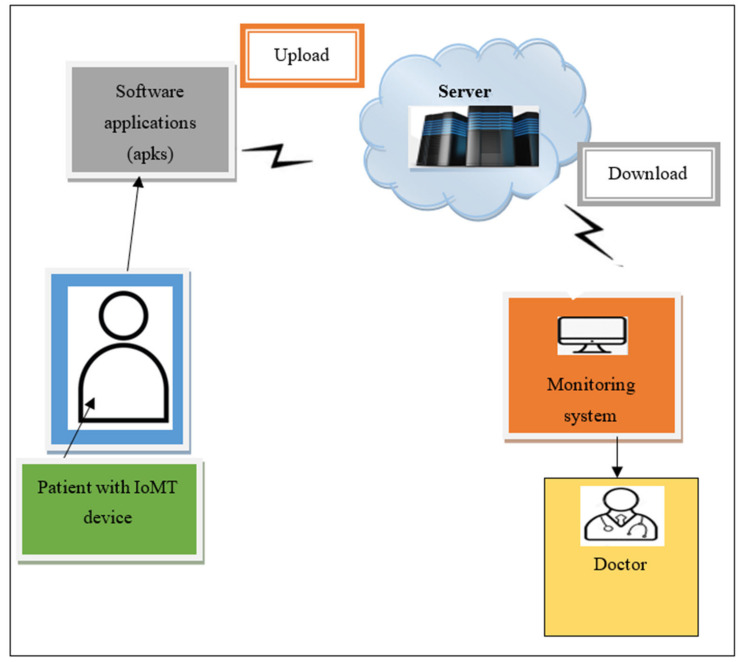
Simple architectural vision of an Internet of Medical Things -based system.

Figure 1 illustrates the simple architectural vision of an IoMT-based system. In general, the “medical things” that act as IoMT devices are small wireless sensors that are fixed on the patient’s body. These wireless sensors are used to gather health-related information such as echocardiogram signals, heart rate, and blood pressure for continuous patient health monitoring. These collected data are processed and transmitted to a hospital database (i.e., a server) and the doctors through wireless communication (i.e., the internet). This sensitive medical information is processed, and doctors make critical decisions based on it. This continuous monitoring process has been increasingly used, but it incorporates two major concerns: the security and privacy of the data that are being collected.

Medical devices are more susceptible to many security threats and attacks than other network devices. Apart from enabling access to sensitive medical data, this application malware destroys the devices and affects the hospital network. Attacks on these connected devices also cause major physical damage. Therefore, the main aim of the research explained in this paper is to underline the need for security research in the field of IoMT and to address security and privacy concerns through the introduction of a novel BCMPB-RIDMPL technique.

**Figure 2:**
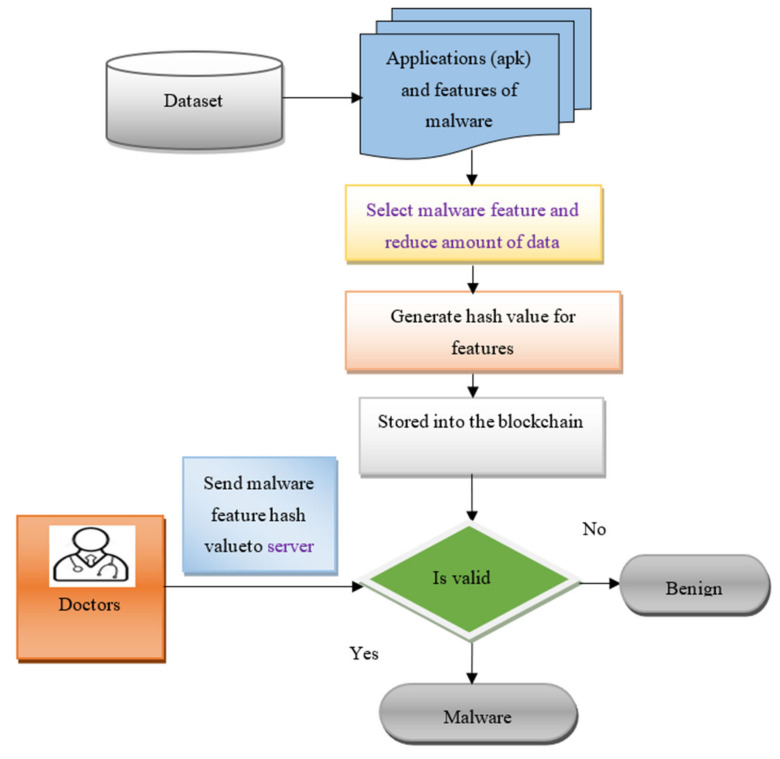
Flowchart of the Biserial Correlative Miyaguchi–Preneel Blockchain-Based Ruzicka-Index Deep Multilayer Perceptive Learning (BCMPB-RIDMPL) technique.

Figure 2 shows the flowchart of the proposed BCMPB-RIDMPL technique to identify the malware and benign applications. The proposed BCMPB-RIDMPL technique firstly selects the malware features to remove unnecessary features in order to reduce the amount of data. This feature information is stored in the blockchain in the form of the hash value. Whenever the doctors in the organization access the patient data, they send the hash value of the malware features to the server. The server verifies these two hash values. Through the use of deep learning, if the hash is valid, the application is classified as malware; if the hash is not valid, the application is classified as benign.

**Figure 3:**
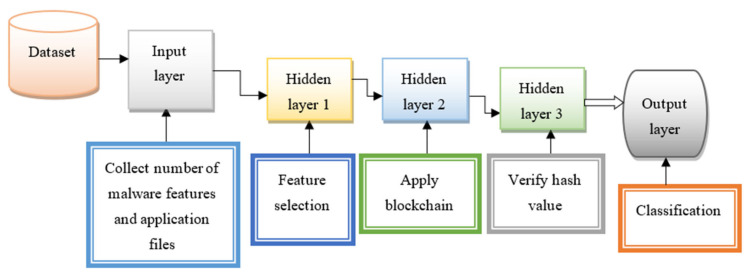
Structure of deep multilayer perceptive learning with one input layer, three hidden layer, one output layer.

The schematic structure of deep multilayer perceptive learning is shown in Figure 3. This method involves neuron-like nodes that are linked in consecutive layers to learn the given input. The layers, in this case, are one input layer, three hidden layers, and one output layer. The output of one layer is linked to the successive layers in a feed-forward manner. The input layer of the network receives the multiple features $A_{1},A_{2},A_{3},\ldots A_{n}$ and applications $f_{1},f_{2},f_{3},\ldots f_{m}$ from the dataset D. The input is transferred into the first hidden layer where the feature selection process is performed.

### 3.1. Point Biserial Correlative Feature Matching

Point biserial correlative feature matching is a statistical technique that is used to choose the relevant features and to remove unnecessary features from high-dimensional space. The mapping process reduces the dimensionality of the datasets. The dataset comprises the malware features $A_{1},A_{2},A_{3},\ldots A_{n}$ in the high-dimensional space. The feature matching finds the relevant feature set based on the correlation measure. The point biserial correlation coefficient is used to measure the correlation between the features in the total set.

The correlation between the features is measured as given in Equation (1):

(1) $$r_{ij}=\frac{\left|A_{i}-A_{j}\right|\sqrt{p\;\left(1-p\right)}}{\sigma }$$

Here, $r_{ij}$ denotes a point biserial correlation coefficient, p denotes a proportion of the feature to select as relevant, and σ denotes a deviation.

(2) $$\sigma =\sqrt{\frac{1}{m}\sum_{i=1}^{n}{\left(A_{i}-\mu \right)}^{2}}$$

In Equation (2), m denotes the number of features, μ denotes a mean value and $A_{i}$ denotes the features. Features that show high correlation are selected and other features are removed. This process helps to reduce the dimensionality of the data and minimize the malware detection time. The selected features are transferred to the hidden layer 2.

### 3.2. Miyaguchi–Preneel Cryptographic Hash-Based Blockchain Technology

**Figure 4:**
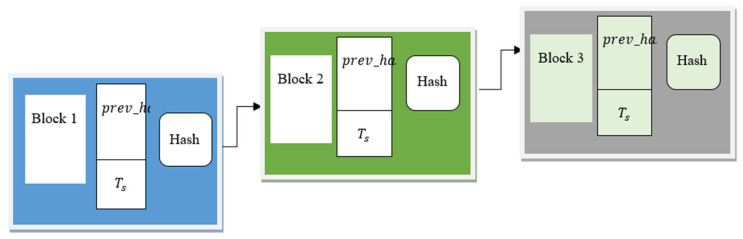
Structure of Miyaguchi-Preneel cryptographic hash-based blockchain for block header, timestamp ($T_{s}$), hash, and a hash of the previous block (prev_hash).

In the second hidden layer, blockchain technology is applied to generate the hash for the improvement of security. Miyaguchi–Preneel cryptographic hash-based blockchain technology is a distributed model that stores the malware features in each block. The basic structure of blockchain technology is shown in Figure 4.

The blockchain comprises a number of blocks that construct a chain. Each block comprises a block header, timestamp ($T_{s}$), hash, and a hash of the previous block (prev_hash). In each block, the hash function of the data takes any length of input data and generates the output with fixed length through the use of the Miyaguchi–Preneel cryptographic compression function. In Figure 4, prev_hash is stored in the current block to ensure that the validation of the block is correct. A similar process is maintained to the end of the blockchain. Each of the previous hash values are combined with the current hash value of the block. The timestamp ($T_{s}$) is used to record the time at which the block was constructed. This helps to track the changes or construction time of the block in a secure manner. 

Miyaguchi–Preneel is a one-way compression function that is used to generate the fixed size of the hash value from the given input data. If there are any changes in the input, these cause severe changes in the hash value. Hence the function is used to guarantee security by avoiding unauthorized access. 

Let us consider the selected features$A_{1},A_{2},A_{3},\ldots A_{k}$. By applying the Miyaguchi–Preneel compression function, the input feature is divided into a number of message blocks of fixed sizes.

(3) $$A\to b_{1},b_{1},\;b_{2},\ldots b_{k}$$

**Figure 5:**
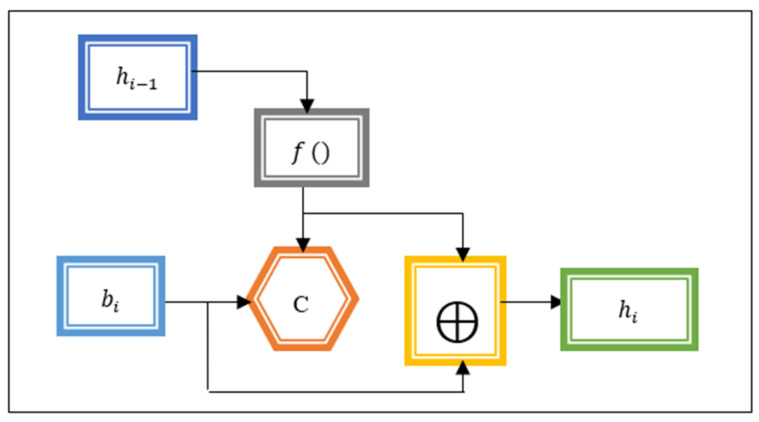
Operation of Miyaguchi-Preneel compression function.

In Equation (3), A specifies an input feature and $b_{1},b_{1},\;b_{2},\ldots b_{k}$ indicates a number of message blocks of a fixed size. The message block is given as input to the Miyaguchi–Preneel compression function to provide the hash value. The operation of the Miyaguchi–Preneel compression function is shown in Figure 5.

Figure 5 portrays the Miyaguchi–Preneel compression function that receives the input message block $b_{i}$ to be encrypted. The previous hash value $h_{i-1}$is fed into the function f () to be converted to fit as the key for block cipher C. The output of the compression function $h_{i}$ is then XOR (⊕) with the previous hash value and the message block ($b_{i}$) as the key to a block cipher. In the first round, the algorithm is set to a constant, pre-specified, initial hash value since there is no previous hash value. The output of the compression function is expressed as follows:

(4) $$h_{i}=[C_{f\left(h_{i-1}\right)}\left(b_{i}\right)⨁\;h_{i-1}⨁m_{i}]$$

In Equation (4), i=1,2,3,…n and $h_{i}$ denotes a hash function. The hash value of one message block is not similar to that of another block, i.e., $h_{1}\neq h_{2}$. The last hash of the message block is taken as the output of the last compression function.

**Figure 6:**
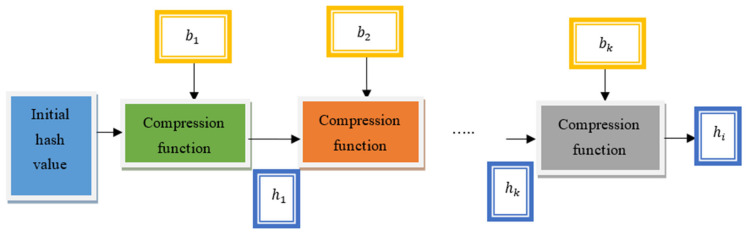
Block diagram of hash generation to generate the hash with the use of the Miyaguchi–Preneel compression function.

Figure 6 illustrates the block diagram for the generation of the hash through the use of the Miyaguchi–Preneel compression function. In this way, a hash value is generated for all the features and the applications to avoid unauthorized access and to increase data security. 

These hash values are stored in the blockchain. The first part of the block header stores the hash values of all data. The second part of the block stores all static and dynamic characteristics of the malware such as suspicious APIs, permissions, events, calls, and so on. This feature information in the block is announced for all the users to predict the malware with higher accuracy and minimum time consumption.

After the information is stored in the blockchain, the classification process to detect malware applications is performed in the third hidden layer. When doctors download the application files, first they verify the hash values of malware features. The hashed information regarding malware features is verified against the test malware features. If these two hash values are matched, then the applications are classified as malware. Otherwise, the applications are classified as benign. The hash value verification is done with the help of the Ruzicka similarity index. 

The Ruzicka similarity is calculated as given below:

(5) $$\varphi_{s}=\frac{h_{F}\cap h_{t}}{\sum h_{F}+\sum h_{t}-h_{F}\cap h_{t}}$$

In Equation (5), $\varphi_{s}$ represents the Ruzicka similarity coefficient, $h_{F}$ represents the hash value of a selected malware feature, $h_{t}$ denotes a hash value of a test malware feature, and $h_{F}\cap h_{t}$ indicates a mutual dependence between the hash values of malware and test features in applications. The Ruzicka similarity function $\varphi_{s}$ provides a similarity value between 0 and 1. The higher the similarity value, the greater the accuracy with which malware is detected. Lower values are classified as benign applications.

The step-by-step process of the proposed BCMPB-RIDMPL technique is shown in Algorithm 1. It is aimed to improve malware detection accuracy through the processes of feature selection, hash generation, and classification. As explained earlier, the deep neural network consists of one input layer, three hidden layers, and one output layer. First, the number of malware features and the applications are provided to the input layer. Then the input is transferred into the first hidden layer to select a number of features from the total features in the dataset based on the correlation measure. Then blockchain technology is applied to generate the hash for each feature and it is stored in the database. Then the verification of the hash values of the testing and training features is performed with the help of the Ruzicka similarity index. Based on the similarity value, the accuracy of malware detection is high. If the two hash values are similar, the application is classified as malware. Otherwise, it is classified as a benign application.
**Algorithm 1: Biserial correlative Miyaguchi–Preneel blockchain-based Ruzicka index deep multilayer perceptive learning.****Input:** Dataset, malware features $A_{1},A_{2},A_{3},\ldots A_{n}$, applications $f_{1},f_{2},f_{3},\ldots f_{m}$ **Output:** Improve the malware detection accuracy**Begin** **Step 1:** Collect the malware features $A_{1},A_{2},A_{3},\ldots A_{n}$, application files $f_{1},f_{2},f_{3},\ldots f_{m}$
**[input layer]**
**Step 2:** For each feature **[hidden layer 1]**
**Step 3:** Measure correlation $r_{ij}$ **Step 4:** Select highly correlated features **Step 5:** Remove other features **Step 6: End for** **Step 7:** Construct blockchain **[hidden layer 2]**
**Step 8: For each** selected feature
**Step 9:** Partition into k message blocks $A\to b_{1},b_{1},\;b_{2},\ldots b_{k}$ **Step 10: For each block **$b_{i}$ **Step 11:** Generate a hash value $h_{i}=[C_{f\left(h_{i-1}\right)}\left(b_{i}\right)⨁\;h_{i-1}⨁m_{i}],$ **Step 12: End for** **Step 13:** Obtain the final hash $h_{i}$ **Step 14: End for** **Step 15:** Store the hash in the blockchain **Step 16:** Perform hash verification **[hidden layer 3]**
**Step 17: Measure similarity **$\varphi_{s}$ **Step 18: If** (arg max $\varphi_{s}$) then **Step 19:** Classified as malware application **Step 20: Else** **Step 21:** Classified as benign application **Step 22: End if** **End**

## 4. Experimental Settings

The proposed BCMPB-RIDMPL technique and two conventional methods, namely the hybrid CNN-LSTM [1] and multi-kernel SVM [2], were assessed through the use of Python by using Drebin dataset. 

### Simulation Setup

A novel Drebin dataset originally developed by the Institute for System Security at the Technical University of Braunschweig, Germany, was used to conduct the experiments to identify malware. This dataset was taken from https://github.com/elsheikh21/malware-analysis (accessed on 9 July 2021). The dataset contains 129,013 application packages, among which 123,453 are benign application packages (.apk) and 5560 are malware android application packages. The dataset comprises comma-separated value (CSV) files. Among the multiple malware features, relevant features were selected for classification. The efficiency of the NGBFSEII-BCP method is determined in terms of malware detection accuracy, the Matthews correlation coefficient, and malware detection time. The hardware specifications are shown in Table 1.

## 5. Evaluation Measures

In this section, the experimental results of the tests on the BCMPB-RIDMPL technique and the state-of-the-art existing hybrid CNN-LSTM [1] and multi-kernel SVM [2] methods are compared with different performance metrics such as malware detection accuracy, the Matthews correlation coefficient, and malware detection time. 

### 5.1. Malware Detection Accuracy

The malware detection accuracy [1] is defined as the number of applications that are correctly classified as benign or malware divided by the total number of applications. The accuracy is formulated as given below.

(6) $$Acc=\left[\frac{TP+TN}{TP+TN+FP+FN}\right]\;*\;100$$

Acc denotes the accuracy, TP denotes the number of true positives,TN is the number of true negatives,FP denotes the number of false positives and FN denotes the number of false negatives. The true positive figure is the number of files that are correctly predicted as malware or benign, while the false positive figure is the number of files that are incorrectly predicted as malware or benign. The accuracy is measured as a percentage (%).

Table 2 shows the results of the experimental analysis of the malware detection accuracy for sequential numbers of applications up to 5000. The Table shows that the detection accuracy of the proposed BCMPB-RIDMPL technique was higher than that of the existing two classifiers. For example, when 500 applications were considered, the use of the BCMPB-RIDMPL technique led to the correct identification of 465 benign or malware packages, which translates to an accuracy of 93%, whereas the malware detection accuracies of the CNN-LSTM and the multi-kernel SVM methods were observed to be 88% and 85%, respectively. In total, ten runs were performed. A comparison of the results shown in Table 2 indicate that the BCMPB-RIDMPL technique achieved an overall detection accuracy that was higher by six percentage points when compared with the CNN-LSTM method and by nine percentage points when compared with the multi-kernel SVM method.

**Figure 7:**
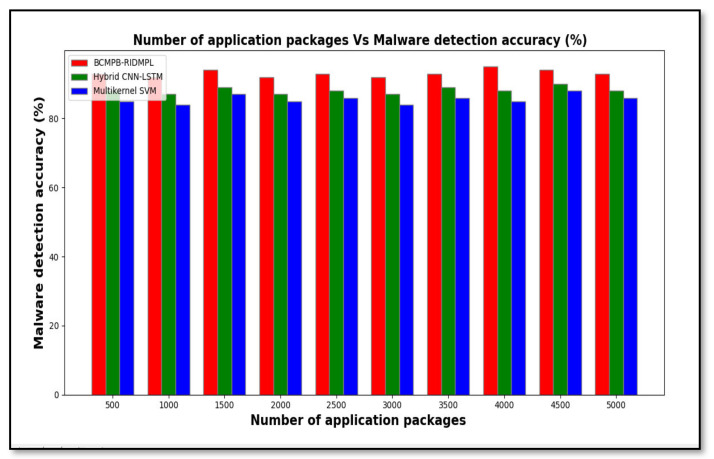
Graphical representation of test of malware detection accuracy for BCMPB-RIDMPL, CNN-LSTM and multi-kernel SVM.

Figure 7 shows the test results from Table 2 in graphical form. The number of application packages is considered in the ranges from 500 to 5000. The number of application packages are taken in the horizontal direction and the malware detection accuracy is observed at the vertical axis. As shown in the graphical chart, there are three various colors of lines such as red, green, and blue indicates malware detection accuracy of three techniques namely BCMPB-RIDMPL, CNN-LSTM and multi-kernel SVM, respectively. Among the three methods, the proposed BCMPB-RIDMPL has the ability to increase malware detection accuracy. The improved detection accuracy that was shown by the BCMPB-RIDMPL technique was achieved by the application of deep multilayer perceptive learning, which employed the Ruzicka index to compare hash values as explained earlier.

### 5.2. Measurement of Matthews Correlation Coefficient

The Matthews correlation coefficient [1] is based on the numbers of application packages that are classified correctly or incorrectly as benign or malware from among the total input. The correlation coefficient is measured using the following formula:

(7) $$MCC=\left[\frac{TP*TN-FP*FN}{\sqrt{\left(TP+FP\right)\left(TP+FN\right)\left(TN+FP\right)\left(TN+FN\right)}}\right]$$

MCC denotes the Matthews correlation coefficient. 

Table 3 displays the Matthews correlation coefficients that were calculated for the BCMPB-RIDMPL technique compared with those of the existing classifiers. The results indicate that the proposed BCMPB-RIDMPL technique outperformed the hybrid CNN-LSTM and multi-kernel SVM methods in terms of the detection of malware. If applied to IoMT devices this improved result would lead to greater prevention of loss or corruption of patient data. The best results for the Matthews correlation coefficient were observed in over 500 applications; the figures were 0.80 for the proposed BCMPB-RIDMPL technique and the CNN-LSTM and multi-kernel SVM methods, 0.76 and 0.74, respectively, followed by various performance results observed for each method. For each method, ten different results are observed. The performance of the proposed BCMPB-RIDMPL is compared to other existing methods. The average Matthews correlation coefficient of these ten sets of results was increased by 19 percentage points and 27 percentage points when compared with the hybrid CNN-LSTM and multi-kernel SVM, respectively.

**Figure 8:**
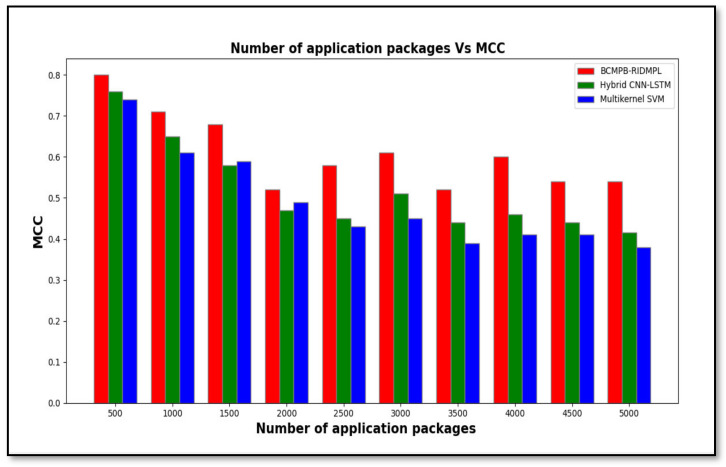
Graph of Matthews correlation coefficient of BCMPB-RIDMPL, CNN-LSTM and multi-kernel SVM method under test.

Figure 8 shows the calculated Matthews correlation coefficients for malware detection by the three methods in graphical form. The chart shows the superior performance of the BCMPB-RIDMPL technique compared with the others. This improvement is achieved through the application of the Ruzicka similarity index-based deep learning concept. Correct matching of the hash values of the test and training malware features leads to their classification as benign or malware applications, and this process reduces the rate of false positives that are reported through the use of the BCMPB-RIDMPL technique compared with the others and improves the accuracy of malware detection.

### 5.3. Malware Detection Time

The time taken to detect malware is defined as the amount of time that passes between data input and detection by the algorithm of benign or malware applications [1]. The overall time consumption is calculated as given below.

(8) $$MDT=m*time\;\left(detectingoneapplications\right)$$

MDT is malware detection time and m denotes the number of applications. The malware detection time is measured in milliseconds (ms).

Table 4 shows the experimental results for malware detection time based on the number of application samples that were collected from the Drebin dataset. It can be seen that the malware detection time increased for all three methods as the amount of input increased. Comparatively, the proposed BCMPB-RIDMPL technique detected the malware in a shorter period than the other methods. Considering 500 applications, the time taken to detect malware or benign applications through the use of the proposed BCMPB-RIDMPL technique was found to be 15 ms, compared with 18 ms and 20 ms for the hybrid CNN-LSTM or multi-kernel SVM systems, respectively. Similarly, ten results are observed for each method. Finally, an average of ten results indicates that the malware detection time is considerably minimized. Comparison of the average results that were observed for the ten input figures shows that the malware detection time through the use of the BCMPB-RIDMPL technique was considerably reduced by eight percentage points and 15 percentage points compared with the existing classifiers.

**Figure 9:**
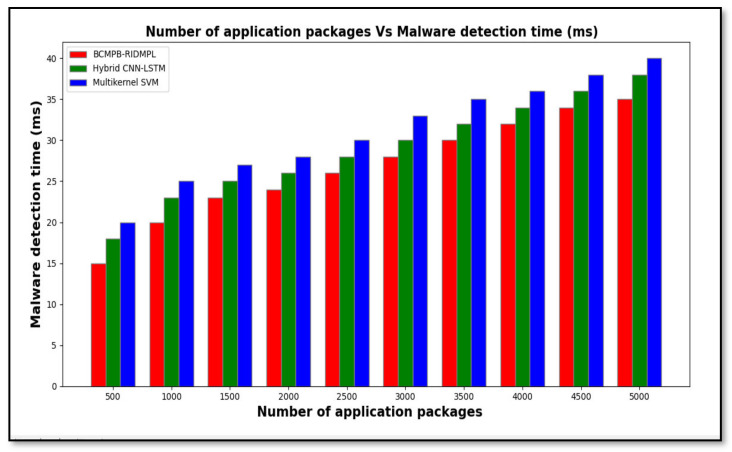
Graphical representation of detection periods of malware for BCMPB-RIDMPL, CNN-LSTM and multi-kernel SVM methods under test.

The shorter detection period shown by the BCMPB-RIDMPL technique, which is displayed graphically in Figure 9, is due to the application of the point biserial correlative feature matching technique to select the relevant features and to remove unnecessary features from high-dimensional space. This mapping process reduces the dimensionality of the datasets. Therefore, the classification is performed with a reduced number of features, which reduces the time taken to complete the malware detection process.

### 5.4. AUC Curve for BCMPB-RIDMPL Technique

The area under the curve (AUC) is a measurement of the performance of the results of the classification of true positives and false positives. A Receiver Operator Characteristic (ROC) curve or the Receiver Operating Characteristic curve refers to the graph indicating the performance of the BCMPB-RIDMPL technique. The AUC-ROC curve is used to solve the binary classification issue. The AUC curve plots two metrics, namely true positives and false positives. Area Under the Curve (AUC) is used to compute the ability of a classifier to distinguish between classes. It is used as a summary of the ROC curve. The ROC curve plot illustrates the relationship between true positives and false positives. The AUC-ROC curve provides a better performance of the model at distinguishing between the positive and negative rate in malware detection. The performance of AUC curve of BCMPB-RIDMPL technique and existing methods hybrid CNN-LSTM or multi-kernel SVM systems is illustrated in Table 5.

**Figure 10:**
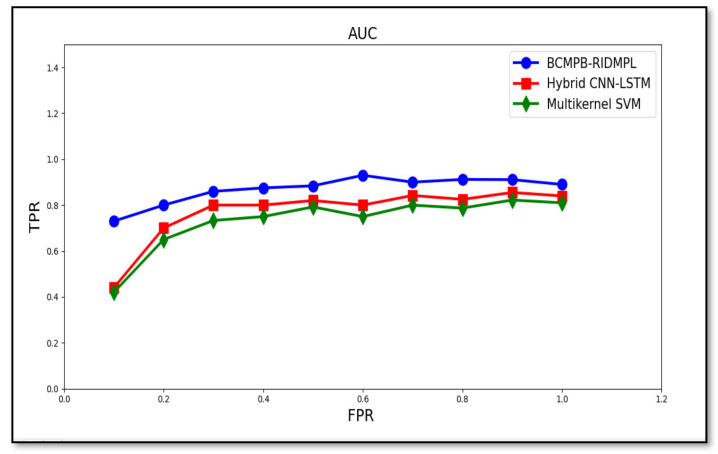
AUC measure of performance of the proposed malware detection technique for BCMPB-RIDMPL, CNN-LSTM and multi-kernel SVM methods.

Performance measurement is an essential task in Machine Learning. AUC-ROC curve [1] is used to handle the output of multi-class classification issues. Figure 10 illustrates the performance of the proposed BCMPB-RIDMPL technique as measured by the AUC for the true-positive and false-positive rates of malware detection. The graph was drawn up for all input results from 500 to 5000. From the results, the AUC-ROC with better TPR was observed using BCMPB-RIDMPL technique upon comparison with existing methods. The reason behind the improvement was due to the application of the Ruzicka similarity index-based deep learning concept.

## 6. Conclusions

The open and widespread environment of the IoMT, which offers high-end digital advantages and involves human lives, creates huge challenges to protect the data from evolving cyber threats, attacks, and vulnerabilities. To address this challenge, we have proposed a highly scalable and efficient malware detection technique. The BCMPB-RIDMPL method initially selects the number of features to reduce the complexity of malware detection. After this feature selection, a hash value is generated through the use of the Miyaguchi–Preneel compression function, and the results are stored in the blockchain. Finally, the Ruzicka similarity index is applied for efficient and timely detection of sophisticated IoMT malware with higher accuracy than is shown by techniques currently in use. A comprehensive experimental estimation of the method has been carried out with consideration of different performance factors such as malware detection accuracy, false-positive rates, and malware detection time. The analysis shows that the proposed BCMPB-RIDMPL technique outperforms the existing classifiers, with higher malware detection accuracy by eight percentage points and the Matthews correlation coefficient by twenty three percentage points, and a shorter detection time by twelve percentage points.

## Figures and Tables

**Table 1:** Hardware Specifications.

Hardware	Specification
Operating system	Windows 10
Processor	core i3-4130 3.40 GHZ
RAM	4GB RAM
Hard disk	1TB (1000 GB)
Motherboard	ASUSTek P5G41C-M
Protocol	Internet

**Table 2:** Comparison of malware detection accuracy.

Number of Applications	Malware Detection Accuracy (%)		
	BCMPB-RIDMPL	Hybrid CNN-LSTM	Multi-kernel SVM
500	93	88	85
1000	92	87	84
1500	94	89	87
2000	92	87	85
2500	93	88	86
3000	92	87	84
3500	93	89	86
4000	95	88	85
4500	94	90	88
5000	93	88	86

**Table 3:** Comparison of Matthews correlation coefficient.

Number of Application Packages	MCC		
	BCMPB-RIDMPL	Hybrid CNN-LSTM	Multi-Kernel SVM
500	0.80	0.76	0.74
1000	0.71	0.65	0.61
1500	0.68	0.58	0.59
2000	0.52	0.47	0.49
2500	0.58	0.45	0.43
3000	0.61	0.51	0.45
3500	0.52	0.44	0.39
4000	0.60	0.46	0.41
4500	0.54	0.44	0.41
5000	0.54	0.416	0.38

**Table 4:** Comparison of malware detection times.

Number of Applications	Malware Detection Time (ms)		
	BCMPB-RIDMPL	Hybrid CNN-LSTM	Multi-Kernel SVM
500	15	18	20
1000	20	23	25
1500	23	25	27
2000	24	26	28
2500	26	28	30
3000	28	30	33
3500	30	32	35
4000	32	34	36
4500	34	36	38
5000	35	38	40

**Table 5:** Comparison of AUC curve.

FPR	AUC Curve		
	TPR		
	BCMPB-RIDMPL	Hybrid CNN-LSTM	Multi-Kernel SVM
0.1	0.73	0.44	0.42
0.2	0.8	0.7	0.65
0.3	0.86	0.8	0.733
0.4	0.875	0.8	0.75
0.5	0.884	0.82	0.792
0.6	0.93	0.8	0.75
0.7	0.9	0.842	0.8
0.8	0.912	0.825	0.787
0.9	0.911	0.855	0.822
1	0.89	0.84	0.81

## Data Availability

Not applicable.
